# Increasing Kidney-Exchange Options Within the Existing Living Donor Pool With CIAT: A Pilot Implementation Study

**DOI:** 10.3389/ti.2023.11112

**Published:** 2023-06-05

**Authors:** Marry de Klerk, Judith A. Kal-van Gestel, Dave Roelen, Michiel G. H. Betjes, Annelies E. de Weerd, Marlies E. J. Reinders, Jacqueline van de Wetering, Marcia M. L. Kho, Kristiaan Glorie, Joke I. Roodnat

**Affiliations:** ^1^ Erasmus Medical Center, Department of Internal Medicine, Transplantation Institute, Rotterdam, Netherlands; ^2^ Department of Immunohematology and Blood Transfusion, Leiden University Medical Center (LUMC), Leiden, Netherlands; ^3^ Erasmus Q-Intelligence, Erasmus University Rotterdam, Rotterdam, Netherlands

**Keywords:** kidney-exchange, AB0-desenzitisation, HLA-desenzitisation transplantation, computerised allocation, highly immunised

## Abstract

Computerized integration of alternative transplantation programs (CIAT) is a kidney-exchange program that allows AB0- and/or HLA-incompatible allocation to difficult-to-match patients, thereby increasing their chances. Altruistic donors make this available for waiting list patients as well. Strict criteria were defined for selected highly-immunized (sHI) and long waiting (LW) candidates. For LW patients AB0i allocation was allowed. sHI patients were given priority and AB0i and/or CDC cross-match negative HLAi allocations were allowed. A local pilot was established between 2017 and 2022. CIAT results were assessed against all other transplant programs available. In the period studied there were 131 incompatible couples; CIAT transplanted the highest number of couples (35%), compared to the other programs. There were 55 sHI patients; CIAT transplanted as many sHI patients as the Acceptable Mismatch program (18%); Other programs contributed less. There were 69 LW patients; 53% received deceased donor transplantations, 20% were transplanted via CIAT. In total, 72 CIAT transplants were performed: 66 compatible, 5 AB0i and 1 both AB0i and HLAi. CIAT increased opportunities for difficult-to-match patients, not by increasing pool size, but through prioritization and allowing AB0i and “low risk” HLAi allocation. CIAT is a powerful addition to the limited number of programs available for difficult-to-match patients.

## Graphical Abstract

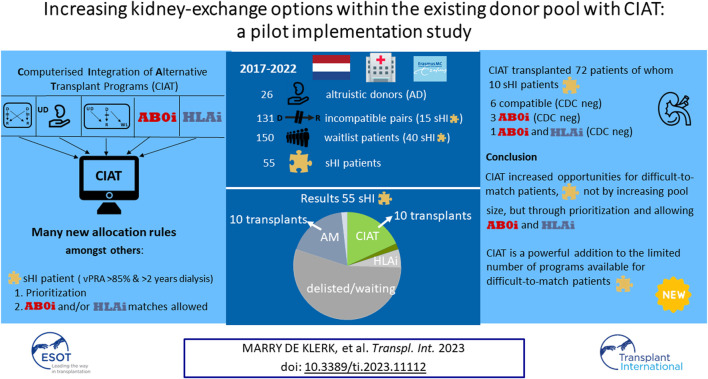

## Introduction

A number of alternative, living donor kidney transplantation programs have been developed for incompatible pairs: Kidney exchange program (KEP), altruistic donor transplantation, domino donation, AB0-incompatible transplantation (AB0i) and HLA-incompatible transplantation (HLAi) [1–11]. In the Netherlands, the national KEP is the only computer based and nationally operating alternative living donor transplantation program. All other programs function locally.

**FIGURE 1:**
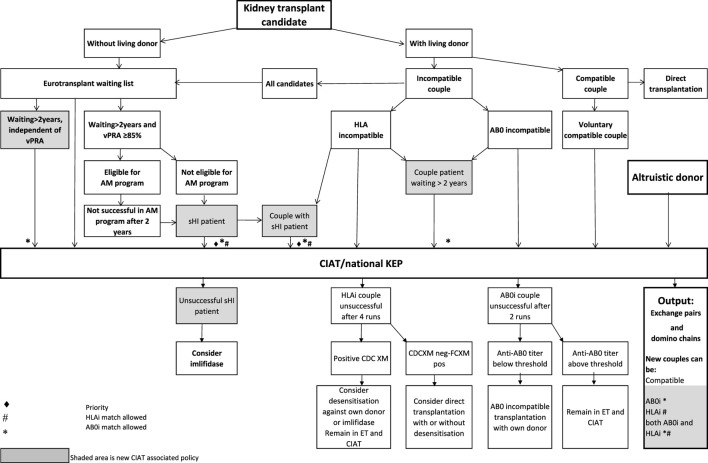
Flowchart to show possibilities to participate in various transplantation programs, for potential transplant candidates with and without a potential living donor.

In the Netherlands, current practice is for incompatible couple recipients to also participate in the deceased donor Eurotransplant waiting list. All incompatible couples are allowed in KEP. After a number of unsuccessful KEP runs, AB0i and/or HLAi couples may opt for desensitization against their intended donor, dependent on anti-AB0-titer and/or donor specific antibody (DSA) level (Figure 1).

Regardless of the presence of a potential living donor, immunized patients can opt for the Eurotransplant Acceptable Mismatch deceased donor program (AM program) after 2 years on dialysis, when vPRA is above 85% (Figure 1). Acceptance in the AM program depends on immunologic criteria [12]. Despite all these programs, many long waiting (LW) and highly immunized patients (HI) do not find a match and accumulate on the waiting list.

Computerized Integration of Alternative Transplantation (CIAT) programs were developed to optimize the kidney exchange program [13]. There are many new options in CIAT compared to current donor-exchange programs [14]. The most eye-catching innovations are integration of altruistic donation, privileges for long waiting (LW) patients, and privileges and priority for a selection of highly immunized (sHI) patients (Figure 1). LW is defined as more than 2 years on dialysis (independent of vPRA): in CIAT, as a privilege, AB0i allocation is allowed for them, provided that AB0 blood type titers are not too high (In our center <1:512, but there are large differences between laboratories). This privilege is introduced because outcomes for living donor AB0i kidney transplantation are superior to waiting for an AB0-compatible deceased donor transplantation [15]. sHI is defined as vPRA ≥ 85% and >2 years unsuccessful participation in the AM program or vPRA ≥ 85% and >2 years dialysis, but is declined for AM on immunologic grounds (Figure 1). The threshold of 2 years AM participation is based on the rapidly decreasing transplant rates with AM after that time point [12]. For highly immunized patients not eligible for AM transplantation, chances are even lower due to lack of priority allocation. All sHI patients are LW patients but when LW patients are upgraded to sHI patients, the LW qualification is no longer used for them. Of course, they keep the privilege to receive an AB0i match (Figure 1).

In CIAT, the chances of sHI patients are increased by giving priority. Matching of sHI patients is the very first step in the CIAT allocation algorithm. Besides, their chances are increased by privileges: acceptance of HLA-incompatible matches are allowed through delisting of HLA-unacceptables with relatively low MFI, unless they are repeated mismatches. This allows “low risk” HLAi allocation to sHI patients. The first aim in the algorithm is a compatible match, then AB0i, then HLAi, subsequently both AB0i and HLAi combinations are aimed at. Transplantation of (highly immunized) patients gives them a survival benefit compared to continuing dialysis [16]. In CIAT, a CDC negative cross-match is mandatory to continue with the transplant in order to prevent breakup of chains because of positive cross-matches. Because altruistic donation is better integrated, waiting list patients can also receive a living donor kidney at the end of a domino chain and privileges, and respectively priority and privileges may also hold for LW and sHI waiting list patients (Figure 1). In the future, when CIAT becomes the national program, the wishes regarding the donors’ donation center can be taken into account. If donors decide to donate in their own center, the domino donors can still participate nationwide.

Currently, worldwide practice is to look for compatible matches first and subsequently, when unsuccessful, accepting incompatible matches requiring desensitization against their intended donor. CIAT is a step in between: it looks for the best CDC negative (in-) compatible match for difficult-to-transplant patients. E.g., CIAT may find a compatible, or AB0i, or CDC-negative HLAi, or both AB0i and HLAi match for a recipient that has a CDC-positive cross-match with the intended donor. CIAT increases chances, not by increasing the available living donor pool size, but by increasing options within the pool. E.g., allowing AB0-incompatible allocation in KEP for a blood type 0 recipient with low anti A and B titers more than doubles the potential donor pool from 47% (only blood type 0) to 100% (all AB0 blood types). Delisting a highly immunized patient’s low-MFI titer unacceptable HLA-A2 allows an HLA specificity that occurs in 30% of the population. The increase in chance depends on the composition of all the patients’ unacceptables being delisted. The more low-titer unacceptables can be delisted, the larger the potential pool and the increase in chance.

In our simulation study we compared results of the national KEP in 2015 and 2016 with those of a CIAT simulation using the same participant input.

Results were very promising [13]. The simulation showed increased match numbers, both overall and in difficult-to-match patients when using CIAT. CIAT found 8 matches for difficult-to-match sHI patients compared to only 1 in reality. In addition, more AB0 compatible (AB0c) matches were found for AB0i couples, while the total number of transplantations was not hampered. Prioritizing difficult-to-match patients improves their chances without affecting the chances of regular patients.

The current study describes the results of CIAT since its implementation in clinical practice over 5 years. The CIAT algorithm was used in one pioneering center, alongside the national KEP and all other alternative transplantation programs, to gain real-life experience. The research question concerns the contribution of CIAT allocation to the total number of transplants, as well as to transplants in long-waiting and highly immunized patients in real-life.

## Patients and Methods

### Patient Data and Ethics

Written informed consent to use their data for research on kidney transplantation was asked and was obtained at the moment patients present for kidney transplantation. The data for this study were retrospectively retrieved from patient files. According to the Dutch law, this study was exempt from approval from an ethics board. Patients and data were treated in accordance with the Declaration of Helsinki and the Declaration of Istanbul.

### CIAT

A local pilot was established in our center between 1st January 2017 until 1st January 2022 to gain logistic experience, to test the algorithm and to optimize the program. Observation was until 1st September 2022. All incompatible couples, compatible couples, and altruistic donors from our center that opted for an alternative donor transplantation program in the period studied, as well as the complete local deceased donor waiting list were included in the pilot.

The additional transplant options of CIAT were tested in the presence of the standard (competing) national and local, deceased and living, donor kidney transplantation programs. There were no exclusions for participation in any program: in the period studied patients participated in all programs available for them. CIAT results were assessed against standard available transplant programs.

#### CIAT: Identification and Handling of sHI and LW Candidates

Participation in CIAT as an LW or sHI patient was discussed and decided by a standing committee. Patients were evaluated on their medical condition in order to determine if there were contra-indications for AB0-desenzitisation. Eligibility criteria for CIAT for sHI or LW patients have been described before and in the introduction [13].

#### CIAT: Match Runs

In this pilot, CIAT operated locally with 4 runs per year, in between national KEP runs. So, in the study period, couples participated every 6 weeks in a match run (taking turns participating in CIAT or national KEP). AB0i, HLAi, and combined AB0i/HLAi couples, as well as (small numbers of) compatible couples and altruistic donors participated in these runs. CIAT can result in both short, closed cycles and open (domino) chains. Closed cycles were formed by 2 or more couples. In domino chains, an altruistic donor started a chain with 1 or more couples, and the donor of the last couple (domino donor) donated to a CIAT selected patient on the waiting list. This might be an sHI or LW waiting list patient. Of the HLA incompatible allocations, only those with a CDC negative cross-match proceeded to transplantation, because desensitization was not allowed in combination with a CIAT match.

During the study period, patients and couples participated in CIAT while also participating in all other available programs.

### Other Alternative Transplantation Programs Available in the Period Studied

Alternative programs are not integrated, as patients participate in all programs separately. The process of finding a match amongst all these programs starts with the search for compatible matches via KEP. When unsuccessful the other programs are tried (Figure 1).

National KEP is the only nationally organized alternative living donor transplantation program: All 7 Dutch transplant centers participate. In national KEP about 3 times as many couples participate per run compared to local CIAT runs. This National KEP runs 4 times per year. AB0i, HLAi, and both AB0i and HLAi couples, as well as small numbers of compatible couples and altruistic donors participate in KEP. Compatible matches in short, closed cycles and open domino-chains are aimed for. In case of a domino paired procedure, the last domino donor is assigned to the transplant center of the altruistic donor. This center selects a waiting list recipient [14]. Current national KEP and CIAT have the same position in Figure 1, but CIAT adds options (shaded areas).

The domino paired donation program starts with an altruistic donor. Together with incompatible couples an open chain is accomplished with the last donor (the domino donor) donating to the waiting list [8]. In the Netherlands this program primarily operates locally.

The AB0-incompatible transplant program is available for AB0i couples that meet the inclusion criteria: anti-AB0 blood type IgG titers 1: 512 and lower [15]. Before proceeding with an AB0i transplant, couples are advised to participate 2 times in KEP.

The HLA-incompatible program is a desensitization program for difficult-to-match, highly sensitized patients with an HLAi living donor [17]. Couples are eligible after unsuccessful participation in the KEP and AM program.

The Eurotransplant Acceptable Mismatch program (AM) for deceased donor transplantation is available since 1989 for highly sensitized patients with vPRA ≥ 85% who are at least 2 years on dialysis [12, 18]. Inclusion depends on immunologic criteria (Figure 1).

## Results

Between January 2017 and January 2022, 946 transplantations have been performed in our center, and 483 with a deceased donor. There were 463 living donor transplantations, of which 338 were direct living donor transplantations and 125 were alternative program transplantations (27% of living donor transplantations). Participants in alternative transplantation programs were: 26 altruistic donors, 131 couples (70 AB0i, 53 HLAi (some of them also AB0i) and 8 compatible pairs). 69 LW and 55 sHI patients participated (Tables 1, 2). Sixteen of these 55 sHI patients had been declined for the AM program. There were 15/55 sHI and 13/69 LW candidates with a potential living donor that participated as a couple. Thus, in total 28/131 couples had a difficult-to-match recipient. On average 150 waiting list patients were included per CIAT run.

**TABLE 1:** Characteristics of LW transplanted according to different programs or still waiting.

	LW patients	LW patients matched in CIAT	LW patient matched in other living donor program	LW patient matched in deceased donor program	LW patient not transplanted
Number	69	14	5	36	14
With a living donor (yes)	13	5	4	1	3
AM program (yes)	11	0	1	3	7
vPRA% (median, range)	22 (0–100)	4 (0–74)	58 (0–100)	4 (0–98)	99 (5–100)
Dialysis vintage (median, range)	3.6 (2–20.8)	3.3 (2–6)	3.1 (2.8–3.2)	3.5 (2.5–9.3)	5 (3.0–20.8)
Bloodgroup: 0	35	6 (2 AB0i)	1	21	7
A	8	5	0	1	2
B	26	3	4	14	5
AB	0	0	0	0	0

**TABLE 2:** Characteristics of sHI transplanted according to different programs or still waiting.

	sHI patients	sHI patients matched in CIAT	sHI patient matched in other living donor program	sHI patient matched in deceased donor program	sHI patient not transplanted
Number	55	10	4	11	30
With a living donor (yes)	15	2	4	2	7
AM program (yes)	39	4	3	11	23
vPRA% (median, range)	99 (85–100)	97 (85–100)	100 (94–100)	99 (88–100)	99.5 (91–100)
Dialysis vintage (median, range)	5 (1.8–23.9)	4.2 (2.1–8.8)	7.5 (3.7–17)	4.1 (2.8–12.2)	5.5 (2.7–23.9)
Bloodgroup: 0	23	3 (1 AB0i, 1 HLAi and AB0i)	1	7	12
A	18	5 (1 AB0i)	2	2	9
B	8	1 (1 AB0i)	1	1	5
AB	6	1	0	1	4

### Transplantations via All Available Programs

**FIGURE 2:**
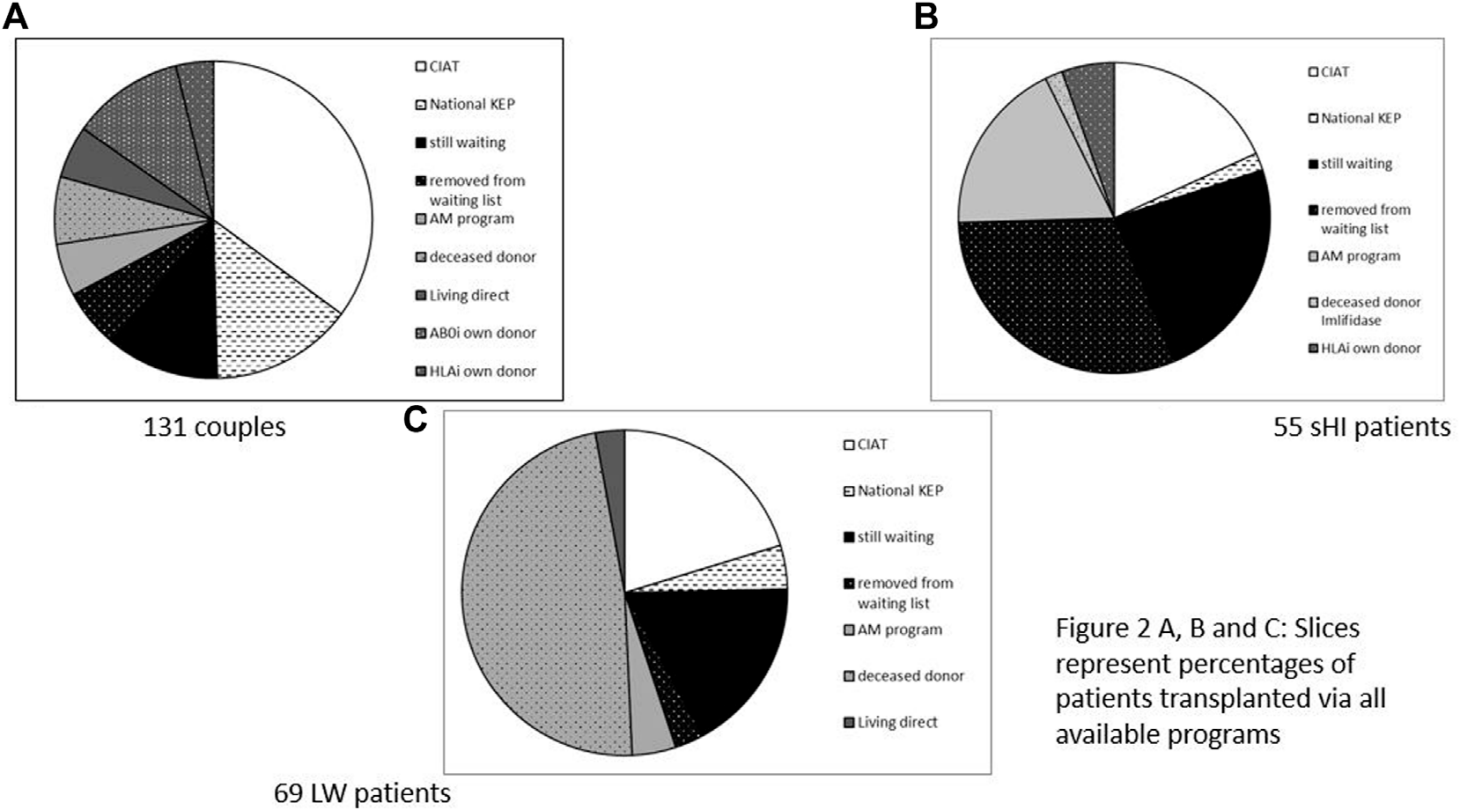
**(A–C)** Slices represent percentages of patients transplanted via all available programs. **(A)** 131 Couples. **(B)** 55 sHI patients. **(C)** 69 LW patients.

*131 incompatible couples* participated (Figure 2A). 46 (35%) were transplanted via CIAT. 27 (21%) received a direct kidney transplantation with another, direct, compatible donor or after AB0 and/or HLA desensitization. 23 (18%) were removed from the waiting list or still waiting, 16 (12%) received a deceased donor kidney, and 19 (14%) were transplanted via national KEP.

There were *55 sHI patients* (Figure 2B), 30 patients (55%) were not transplanted. Ten patients were transplanted via CIAT (18%), while another 10 (18%) received an AM deceased donor kidney, one patient received a HLAi deceased donor kidney after Imlifidase desensitization (2%). There were 3 (5%) direct living donor transplantations via HLA desensitization and 1 (2%) via national KEP.

There were *69 LW patients* (Figure 2C) of whom 14 (20%) were transplanted with a living donor kidney via CIAT; 14 (20%) were removed from the waiting list or still waiting; 36 (52%) received a deceased donor kidney (including AM), and two patients received a compatible living donor kidney via direct donation and three via national KEP (7%).

### Transplantations via CIAT

In total 72 transplantations have been performed via CIAT (Table 3). The majority was transplanted in an open cycle starting with an altruistic donor.

**TABLE 3:** CIAT match and transplant results in the pilot period.

2017–2022				
Number	Cycle/chain	Pair	Waiting list patient	Transplant
6	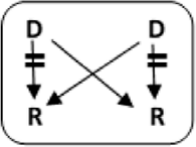	12		12
2	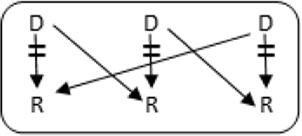	6		6
1	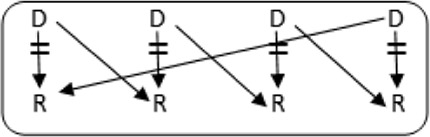	4		4
12	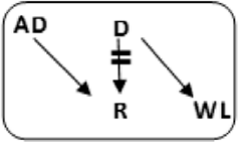	12	12	24
6	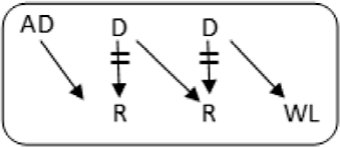	12	6	18
8	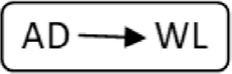		8	8
Total		46	26	72

AD, anonymous/altruistic donor.

In total, 46/131 couples were matched via CIAT: 30/70 AB0-incompatible couples (43%), and 10/53 HLA-incompatible couples (19%); 5 in a closed chain and 5 in an open cycle starting with an altruistic donor. Six of the eight compatible pairs were matched. All matches resulted in donation and transplantation. Two compatible pairs were not matched after one CIAT run, and they decided on direct donation and transplantation.

From the 72 patients transplanted via CIAT, 14 were LW patients, of whom 2 patients received an AB0i transplant (Table 1). Ten were sHI patients, of whom nine received an HLA compatible transplant, and three were AB0i. One sHI patient received an AB0i and HLAi transplant (Table 2). This latter patient was transplanted with a donor kidney against whom he had low titer HLA unacceptables. CDC cross match was negative. Tables 1, 2 shows details and characteristics of the patients transplanted or not in the various programs.

Table 4 shows characteristics and long-term outcomes of 10 CIAT transplanted sHI patients. It is a relatively young population with high vPRA and long dialysis time. Most of them received a retransplant. For 2 with a potential living donor, CIAT found a closed chain. Only 3 sHI patients received their kidney directly from the altruistic donor, all others were in a chain. Observation time is between 6 months and more than 5 years. One patient had a never functioning graft because of recipient comorbidity and rejection. Another failed after 6 months because of CMV reactivation, BK nephropathy and rejection. Transplant function of the other patients is acceptable to good (Table 4).

**TABLE 4:** Characteristics and long-term outcome of 10 via CIAT transplanted sHI patients. Patient 10 received an AB0i and HLAi transplant with a negative CDC cross-match.

Patient nr	Age	Gender	vPRA	Time on dialysis (years)	Previous kidney transplants	Potential living donor	AB0 combination D->R	Type chain	HLAmm	Time after transplant (years)	Rejection therapy	eGFR mL/min/1.73 m^2^
1	73	M	98	2.7	1	n	A->A	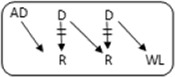	1-1-0	5.1	N	39
2	39	F	100	8.8	3	y	***B->A***	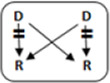	1-1-0	0.0	Y	NFG
3	46	F	85	2.7	1	n	A->A	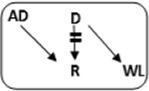	1-2-2	4.3	N	40
4	48	F	99	4.9	0	n	***A->O***	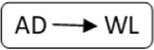	0-1-2	3.4	N	31
5	72	F	91	6.7	0	n	0->0	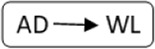	2-1-1	2.5	N	87
6	25	F	96	4.6	1	y	***A->B***	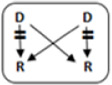	2-2-0	2.4	Y	70
7	53	M	100	2.1	5	n	0->A	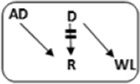	1-1-0	2.8	N	76
8	63	F	92	2.4	0	n	A->AB	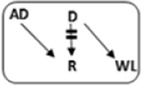	1-1-1	2.6	N	35
9	32	F	99	3.8	2	n	A->A	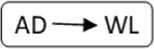	1-1-1	0.8	N	46
10	40	M	94	8.6	1	n	***A->0***	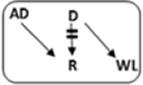	1-1-2	0.6	Y	Failed

Bold-italic values are the blood type incompatible combinations between donor and recipient.

Ten other sHI patients received a kidney via the AM program, observation was between 0 and 4 years. Two kidneys failed 0.3 and 3 years after transplantation. One patient died 0.2 years after transplantation. eGFR of the remaining seven functioning kidneys is between 29 and 99 mL/min/1.73 m^2^.

## Discussion

In the present study the implementation of additional allocation rules in a kidney exchange program was tested in one center. We compared the performance of CIAT with that of all other local and national, living and deceased donor transplantation programs available for these patients. In our 5-year pilot, CIAT allocation resulted in high numbers of transplantations in HI patients and incompatible couples in comparison to all other programs. Local CIAT even outperformed national KEP on numbers transplanted. CIAT is a major addition to the limited number of existing programs that enable kidney transplantation in difficult-to-match patients. There are no publications on a program like CIAT that integrate KEP, altruistic donation, AB0- and/or HLA-incompatible allocation and transplantation while giving priority and privileges to difficult-to-match patients. The strength of CIAT is that rules and regulations guarantee increased chances given to all these selected patients. CIAT is a new and promising program that is currently being adapted to national requirements in order to replace National Dutch KEP.

In our recent CIAT simulation we showed that, compared to the old situation, the adaptations to KEP led to 8 times more transplantations in HI patients while the total number of transplantations performed was not hampered [13]. Furthermore, far more matches were found for AB0i couples.

In the present pilot only 1 both AB0i and HLAi match was found with a negative CDC cross-match. Presently only CDC negative matches proceed to transplantation to prevent last minute declines because of positive CDC cross-match. Including a patient with a CDC positive cross-match in a chain or cycle is felt to be too complicated because unsuccessful desensitization may lead to disintegration of the complete chain or cycle. One HLAi combination with positive CDC cross-match has been declined in the period studied.

Accumulation of difficult-to-match patients on the waiting list is a universal problem [19–22]. There have been several efforts to solve this problem:

First: In Europe, the AM deceased donor program was introduced in 1989 for difficult-to-match patients, and is based on the positive identification of acceptable antigens [23]. This approach has led to significantly decreased waiting times for highly sensitized patients in the Eurotransplant region [12]. Until now the AM program was the most important program to transplant HI patients. Our pilot shows that even local CIAT can transplant a comparable number of sHI patients. A national CIAT program with a larger pool will likely result in higher transplant rates. In the United States, in 2013 a successful new Kidney Allocation System (KAS) was introduced with priority for highly sensitized patients in the regular deceased donor kidney allocation system [24, 25].

Second: In the direct living donor kidney transplantation population, blood type A and AB recipients are far more easily transplanted compared to blood type B and 0 recipients. The chances of finding a direct living donor for highly sensitized patients are low [21]. This led to the introduction of the aforementioned KEP program, but also to AB0-incompatible and HLA-incompatible transplantation programs. KEP is the backbone of living donor kidney transplantation for incompatible couples and various adaptations to the basic program have been performed and simulated in order to improve the success rate of the program for all participants, and for those difficult-to-match.

Third: Participation of compatible pairs in KEP was studied in simulations and in reality and appeared to improve the chances of difficult-to-match patients by enlarging the pool and adding blood type 0 donors [26, 27]. However, ethical issues like the definition of benefit for the compatible pair needs to be faced [28]. In spite of that, in our present study, compatible couples were successfully included.

Fourth: Another way to improve KEP results might be to expand the pool by international collaboration [29–31]. For collaboration a mutual, international protocol and agreement on the language of instruction are mandatory. Different countries, however, hold different laws and regulations. A simulation showed that, when countries are allowed to have different constraints and goals with regard to the cycles and chains, this may lead to a large discrepancy between the number of participating couples compared to the number of successful matches per country [32]. Another problem is that couples willing to participate in an international program likely will be those very difficult to match. This is reflected by the study by Valentin et al. were 71% of participating patients had vPRA > 80% [30]. If we consider the above-mentioned complexity that international KEP is confronted with, there are still many hurdles and barriers to be taken [33].

However, difficult-to-match patients from one country may benefit from participation to donation programs in another country with a slightly different HLA-pool. In a simulation Mumford et al. showed that highly sensitized patients have modestly increased chances of a match in a different European deceased donor pool [34]. Individual participation of very difficult-to-match patients in foreign living or deceased donation programs could be successful and less complicated.

Finally: As we demonstrated in our current pilot study, an easier way to improve access to transplantation for difficult-to-match candidates is to make adjustments to the current KEPs. The adaptations we studied were priority and privileges for sHI and privileges for LW candidates. This increases options within the same donor pool. In our recent simulation and in the present pilot we demonstrated the benefit for difficult-to-match patients with this approach [13]. Adaptations to other existing KEPs in simulations suggest better results when AB0 incompatible matching is allowed [35, 36]. Real-life AB0-incompatible matching in a kidney-exchange program was allowed in a small-scale Australian study and showed promising results [37]. Integration of KEP and desensitization programs has been attempted temporarily and on a small scale by [38]. CIAT is the first program that combines the possibilities and benefits of different alternative transplantation programs in a kidney exchange program. In the present study, we showed that adaptations to a regular KEP program indeed lead to higher transplantation rates for difficult-to-match patients. However, not only the algorithm is responsible for this success: Rules, regulations, and agreements concerning priority and privileges for selected patients are indispensable. Just by giving priority, 6 sHI patients received a completely compatible match. By combining priority and allowing AB0i and HLAi allocation for sHI patients, a larger part of the potential donor pool becomes available which further increases their chances: another four received an incompatible transplant. In the period studied, CIAT found as many matches for sHI patients as the AM program: both 10 transplantations. CIAT enables transplantation of difficult-to-match patients, even in small pools. We performed 72 transplantations via CIAT, of whom only six patients were allocated an incompatible transplant: four sHI and two LW patients. All others were completely compatible matches.

In conclusion: In spite of current programs that aim at reducing inequality in transplant numbers for difficult-to-match patients, sHI and LW candidates still accumulate on the waiting list. Modifying the algorithm and prioritizing the sHI patients, while allowing AB0-and/or HLA-incompatible allocation, resulted in increased transplant numbers in this population. The participation of altruistic donors is essential as “fire starters” and to enable the participation of waiting list patients. Easy-to-match incompatible pairs and compatible pairs are essential for success because, in order to complete a puzzle, both the difficult and the easy pieces are indispensable. CIAT is a new and welcome addition to existing programs in matching difficult-to-match kidney transplant candidates.

## Data Availability

The data analyzed in this study is subject to the following licenses/restrictions: Data are available on request. Requests to access these datasets should be directed to Marry de Klerk, marry.deklerk@erasmusmc.nl.

## Abbreviations

AB0c, AB0 blood type compatible; AB0i, AB0 blood type incompatible; AD, altruistic donor (altruistic living donor not directed to a specific recipient); AM, acceptable mismatch program from Eurotransplant; CDC XM, complement dependent cytotoxicity cross match; CIAT, computerised integration of alternative transplantation programs; cPRA, calculated panel reactive antibody; DSA, donor specific antibody; ET, Eurotransplant; FCXM, flow cytometry cross match; HI, highly immunised; HLAi, HLA incompatible: positive cross-match; KAS, kidney allocation system; KEP, kidney exchange program; LSA, LABScreen^®^ Single Antigen One Lambda; LW, long waiting; MFI, mean fluorescence intensity; sHI, selected highly immunised; vPRA, virtual panel reactive antibody.
